# Development and validation of a quantitative method for the enumeration of *Salmonella enterica* serovar Infantis from environmental poultry feces based on most probable number approach followed by confirmatory qPCR

**DOI:** 10.3389/fmicb.2026.1861550

**Published:** 2026-06-18

**Authors:** Matteo Ricchi, Rebecca Pezzolato, Anita Filippi, Elena Fabbrocino, Letizia Mantovani, Simona Perulli, Letizia Cirasella, Andrea Luppi, Giovanni Tosi

**Affiliations:** 1 Sede Territoriale di Piacenza, Istituto Zooprofilattico Sperimentale Lombardia Emilia Romagna, Piacenza, Italy; 2 Sede Territoriale di Forlì, Istituto Zooprofilattico Sperimentale Lombardia Emilia Romagna, Forlì, Italy

**Keywords:** environmental samples, ISO 6579-2: 2012, qPCR, quantification, *Salmonella* Infantis, most probable number

## Abstract

*Salmonella enterica* serovar Infantis has emerged over the past two decades as the dominant *Salmonella* serovar in European Union (EU) broiler production. Due to largely asymptomatic colonization in poultry, multidrug-resistant (MDR) strains can disseminate silently within flocks, leading to contamination of meat products and raising significant One Health concerns. In Italy, *S. infantis* represents up to 90% of *Salmonella* isolates from broilers chickens and approximately 40% of human cases, highlighting the need for improved monitoring tools to support risk management strategies. Quantitative microbial risk assessment (QMRA) approaches require accurate and reliable quantitative data. To this end, we developed and validated a rapid and specific method for quantifying *S. infantis* in poultry environmental fecal samples. The method is based on a miniaturized most-probable-number (MPN) approach adapted from ISO 6579-2, combined with confirmation by a *S. infantis*-specific qPCR assay. Matrix effects associated with environmental poultry feces were evaluated using the Bland–Altman approach. Method performance, including repeatability and reproducibility, was assessed using both spiked and naturally contaminated samples. Specificity was ensured by the high analytical specificity of the confirmatory qPCR. When applied to naturally contaminated samples, the method demonstrated suitability for quantifying *S. infantis* loads in poultry environmental feces, providing robust data to support QMRA-based interventions and surveillance strategies within broiler production systems.

## Introduction

*Salmonella enterica* serovar Infantis (*S. infantis*) has emerged as a critical concern in the global poultry industry over the past two decades (Alvarez et al., 2023; Mattock et al., 2024). Once considered a minor serovar., *S. infantis* has undergone a dramatic epidemiological shift, becoming one of the most prevalent serovars associated with poultry production worldwide (Alvarez et al., 2023; Piña-Iturbe et al., 2026). *S. infantis* now represents the dominant serovar in broiler chickens across the European Union (EU) (Petrin et al., 2024), ranking among the top four serovars across all food-animal sources (European Food Safety Authority (EFSA) and European Centre for Disease Prevention and Control (ECDC), 2025) while in the USA is considered still and emerging serovar (Betiku et al., 2025). The concern surrounding *S. infantis* extends beyond its high prevalence. In fact, *S. infantis* has demonstrated a remarkable capacity for acquiring and maintaining multidrug resistance (MDR) through the widespread circulation of the pESI-like megaplasmid (Alvarez et al., 2023; Piña-Iturbe et al., 2026). This plasmid carries multiple antimicrobial resistance genes, including extended-spectrum beta-lactamase (ESBL) determinants, conferring resistance to critically important antimicrobials (Alba et al., 2020). The plasmid-mediated resistance not only compromises treatment options for human infections but also poses significant challenges for disease control in poultry production systems, as resistant strains can persist in flock environments and spread throughout the production chain.

The public health implications are substantial. *Salmonella* spp. in general, including *S. infantis*, can colonize poultry asymptomatically, making detection difficult while allowing silent amplification throughout the production chain (VanderWaal and Deen, 2018). As a consequence, the bacterium’s ability to contaminate meat products during processing creates a persistent risk of foodborne illness in consumers. In this contest, the documented transmission of MDR *S. infantis* from poultry to humans through the food chain underscores the One Health implications of this pathogen (Piña-Iturbe et al., 2026). High environmental contamination with *S. infantis* in broiler chickens farms represents a risk for flock colonization (Gole et al., 2014; Papić et al., 2026) and may consequently increase the likelihood of contaminated carcasses entering the food chain (Nógrády et al., 2008), posing a serious risk for food chain production (Nógrády et al., 2008; Proietti et al., 2020).

The most recent EFSA report indicates that the recovery of *S. infantis* in EU countries is almost exclusively associated with the broiler source (96.5% of isolates of this serovar) (European Food Safety Authority (EFSA) and European Centre for Disease Prevention and Control (ECDC), 2025). However, the report also highlights that these data should be interpreted with caution, as the distribution of serotyped isolates across sources is highly unbalanced. Among isolates from the laying hens/eggs source (*n* = 1,413), *S. infantis* ranked as the fourth most frequently reported serovar and was reported by 13 EU Member States, showing a relatively balanced distribution across reporting countries. In the broiler source (*n* = 15,717), three serovars (*S. infantis*, *S. enteritidis*, and *S. Mbandaka*) accounted for 69% of the serotyped isolates, with *S. infantis* representing more than half of the isolates reported (57.9%). Notably, a marked increase in *S. infantis* isolates was observed in 2024 (9,428), compared with 2023 (7,665) and 2022 (6,840), and these were mainly reported by Italy (European Food Safety Authority (EFSA) and European Centre for Disease Prevention and Control (ECDC), 2025).

According to some authors (Proietti et al., 2020), in Italy, *S. infantis* has been frequently reported in human cases (39.4%), as well as in food and animal sources (European Food Safety Authority (EFSA) and European Centre for Disease Prevention and Control (ECDC), 2025). Furthermore, cross-sectional studies conducted in broilers at slaughter showed that *S. infantis* accounted for 75 and 90% of all *Salmonella* isolates detected in 2014 and 2016, respectively (Proietti et al., 2020).

Recent reviews in microbiological risk assessment emphasize the central role of quantitative microbial risk assessment (QMRA) frameworks, where numerical inputs and probabilistic modelling allow explicit estimation of exposure, dose–response relationships, and risk outcomes, offering greater transparency and utility than purely qualitative assessments (de Carvalho et al., 2025; van der Vossen-Wijmenga et al., 2025). In the context of food and water safety, quantitative risk assessments use numerical data at every stage—hazard identification, exposure estimation, and risk characterization—enabling decision-making based on probability distributions rather than categorical descriptors, a key advantage over qualitative approaches (Clements et al., 2025).

With the objective of generating robust and precise data to support the optimization and management of intervention strategies aimed at mitigating the risk of *S. infantis* within the food chain, we have developed and validated a rapid, highly specific analytical tool for the quantification of *Salmonella enterica* subsp. *enterica* serovar Infantis in poultry environmental fecal samples.

## Materials and methods

To enable the detection and quantification of *Salmonella* Infantis in poultry environmental fecal samples, we developed and validated an integrated protocol combining molecular and culture-based microbiological approaches, thereby ensuring both reliable identification and accurate quantification of the bacterial load.

In more details, the procedure was focused on the Most Probable Number (MPN) method, a statistical approach applied to serial sample dilutions which allows the quantification also of low loads of *S. infantis* (Jarvis et al., 2010). In more details, the quantification followed the UNI CEN ISO/TS 6579–2:2012 standard, but confirmation of *S. infantis* in each positive well was performed using Real-Time PCR (qPCR), taking advantage of its high specificity to detect the target organism even in complex, microbially rich matrices.

Validation was carried out using *S. infantis* negative poultry fecal samples previously confirmed. The whole procedure takes 3 days.

### Method of quantification

The first step consisted of a pre-enrichment step to resuscitate stressed or injured *Salmonella* cells that may be unable to grow directly on selective media. Briefly, 1 g of avian feces was resuspended in 9 mL of Buffered Peptone Water (BPW) and vortexed for 15 s to release bacteria from the fecal matrix. An aliquot (0.25 mL) of the suspension was aseptically transferred into the first column of a 24-well plate containing 1 mL BPW per well. Serial five-fold dilutions were then performed to obtain six dilution levels, each prepared in four replicates. Plates were incubated at 37 ± 1 °C for 18 ± 2 h under non-selective conditions to allow recovery and initial multiplication of *Salmonella* spp. cells prior to selective enrichment.

After this step, 0.02 mL of each well is transferred to another 24 plate well previously filled with 1 mL of selective medium, the Modified Semi-solid Rappaport-Vassiliadis medium (MSRV) and then incubated for 24 h ± 2 h. After this incubation period, only cleared wells, defined according to UNI CEN ISO/TS 6579–2:2012 as those in which *Salmonella* spp. (including *S. infantis*) could have developed, were considered for the subsequent step of the method. Notably, these two steps are those reported within the UNI CEN ISO/TS 6579–2:2012.

The final stage involves confirming the presence of Salmonella Infantis in each tested well. Briefly, a small aliquot (approximately 10 μL) of clarified culture medium, indicative of microbial growth, is collected using a sterile metal loop and transferred into tubes containing 1 mL of distilled water supplemented with 5 μL of internal control (qPCR Extraction Control Red Internal Control DNA, Meridian Bioscience, Cincinnati, OH, USA). The tubes were boiled at 98–100 °C for 10 min and centrifuged (12,000 g for 2 min). Five μl of the supernatant previously obtained were added in each qPCR well. Quantitative PCR was run in 20 μL final volume in a CFX 96 thermal cycler (Bio-rad, Milan, Italy) with the following thermal profile: 95 °C for 2 min denaturation/activation of the TAQ polymerase, 95 °C for 15 s, denaturation, annealing/extension 60 °C for 60 s, for a total of 40 cycles. The GoTaq® Probe qPCR Master Mix (Promega, Madison, WI, USA) was used, with primer and probe concentrations of 300 nM and 150 nM, respectively. The primer sequences were adopted from Yang et al. (2020), while the probe sequence was manually designed as FAM 5′-TCTTAGGTGAACGCAT-3′-BHQ1. For each qPCR run, a negative extraction control, a negative amplification control, and a positive amplification control—consisting of previously extracted *S. infantis* DNA—were included.

Wells with a PCR Cq value <37 were considered positive for *S. infantis* and, consequently, were used for enumeration using the Excel calculator sheet provided by UNI CEN ISO/TS 6579–2:2012 to determine *S. infantis* counts per gram of environmental poultry feces. Notably, since the software used (MPN calculation program, version 6, dated 2018-11-07; original paper: Jarvis et al., 2010) also allowed the identification of unlikely combinations, results associated with combinations whose probability did not fall within the acceptable category (those defined by a rarity score of 3) were considered invalid and the analysis was repeated. Moreover, the calculator sheet employed also included a tool for estimating uncertainty (confidence limits). Specifically, each MPN/g value was accompanied by the 95% confidence limits. This interval does not represent an error but statistically defines the range within which the actual load of Salmonella Infantis in the sample is expected to fall.

### Validation of the method

#### Effect of the fecal matrix on the verification of the minimal analytical sensitivity and limit of quantification (LOQ) by Bland and Altman approach

The quantification method described in this study is adapted from the ISO/TS 6579–2:2012 protocol, with modifications primarily due to a reduction in the sample amount tested. Quantification using the MPN (Most Probable Number) approach is based on the number of replicates that test positive for the presence of the pathogen and the dilution level at which positive results are no longer observed. In addition, the proportion of matrix processed in the assay contributes to the calculation. Using four replicates per dilution across six dilutions (with a dilution factor of five) and a mixture volume of BPW and feces at a 10:1 ratio (0.25 mL), the theoretical minimum limit of detection (LOD) was calculated to be 8.9 MPN/g of feces (or 0.95 log10 MPN/g), based on the Excel tool provided with UNI CEN ISO/TS 6579–2:2012. The minimum detectable concentration corresponds to one positive well out of four at the highest dilution tested. Since this concentration is quantifiable with the Excel tool, the LOD coincides with the limit of quantification (LOQ) for the method.

However, the influence of the matrix can potentially impact both LOD and LOQ. To assess whether the fecal matrix could have an inhibitory effect on the limit of quantification, 1 g of negative environmental poultry feces, repeatedly tested negative using the qualitative method described in ISO 6579-1:2017, was spiked with various dilutions containing from 5–10 to 1,000–10,000 CFU (corresponding to one to three log₁₀) of *S. infantis*. To confirm the actual amount of *S. infantis* used for spiking, the same dilutions were processed using the same method, and results with and without poultry feces were compared using the Bland–Altman approach. Nine experiments, comprising a total of 33 records, were included in this analysis. Notably, because bacterial loads are typically expressed as log₁₀, the results were reported as log₁₀. Records with an absolute value of zero could not be transformed to log₁₀, reducing the number of records available for Bland–Altman analysis of log₁₀-transformed data to 25. To evaluate the effect of matrix in the LOQ, *S. infantis* NCTC 6703/IZSLER 2150 reference strain (four experiments for a total of 16 records) and two previously recovered field isolates were used. Bland–Altman analysis was performed using SigmaPlot version 13.0 (Grafiti LLC).

#### Repeatability and reproducibility

Repeatability and reproducibility were evaluated, according to the “Validation of diagnostic assays for infectious diseases of terrestrial animal”, chapter 1.16 of the WOAH Manual of Diagnostic Tests and Vaccines for Terrestrial Animals, in order to ensure stable results despite the complexity of the fecal matrix.

Repeatability was evaluated by analyzing the same sample in parallel under identical experimental conditions, using the same operator. Repeatability was conducted by analyzing a contaminated fecal sample in duplicate (REP. 1 and REP. 2) at three different concentration levels (ranging from one to three log_10_). Moreover, the same experiment was run using naïve contaminated samples at different concentrations, taking care of selecting also samples containing low concentrations (around one log_10_).

Reproducibility was assessed by applying the same method to the same sample under variable conditions, specifically involving two different operators, demonstrating that the accuracy of the quantitative data is independent of individual manual skills and reflects the intrinsic robustness of the protocol.

Since the method herein developed and validate is a quantitative method, the degree of dispersion of the result was evaluated as coefficient of variation (ratio between standard deviation and mean).

#### Quantitative PCR specificity

According the “Validation of diagnostic assays for infectious diseases of terrestrial animal”, chapter 1.16 of the WOAH Manual of Diagnostic Tests and Vaccines for Terrestrial Animals, both inclusivity and exclusivity of the qPCR assay employed in this method were evaluated. The analytical specificity of the assay relies on the specificity of the primers and probe used in the qPCR. Although the primers were originally designed by (Yang et al., 2020) through the comparison of a large number of serovars, resulting in a primer set specific for *S. infantis*, their inclusivity was further evaluated in this study. Specifically, 29 field isolates of *S. infantis* collected during routine diagnostic activities, along with one reference strain (NCTC 6703/IZSLER 2150), were tested. Exclusivity was evaluated using 60 field isolates of *Salmonella enterica* subsp. *enterica* representing different serovars, along with several non-*Salmonella* bacterial strains, including members of the Enterobacteriaceae family (see Table 1 and Supplementary material).

**Table 1:** Repeatability of spiked **(A)** and naturally contaminated samples **(B)**.

(A) Spiked samples					
Concentration (MPN/g feces)	Rep 1 (log_10_ MPN/g)	Rep 2 (log_10_ MPN/g)	Mean	SD	CV (%)
3 log_10_	2.63	2.99	2.81	0.25	8.9
2 log_10_	1.82	1.94	1.88	0.08	4.3
1 log_10_	1.30	1.23	1.27	0.05	3.9

Real samples					
**Samples**	Rep 1 (log_10_ MPN/g)	Rep 2 (log_10_ MPN/g)	Mean	SD	CV (%)
1	3.95	4.23	4.09	0.20	4.9
2	3.20	2.93	3.07	0.19	6.2

Quantitative PCR (qPCR) efficiency was evaluated using genomic DNA obtained from single subcultures of the reference strain *S. infantis* NCTC 6703/IZSLER 2150 and one field isolate. Briefly, a few colonies were suspended in bidistilled water, and DNA was extracted using the DNeasy Blood and Tissue Kit (Qiagen, Milan, Italy) according to the manufacturer’s instructions. DNA concentration was adjusted to 10 ng/μL and subjected to 10-fold serial dilutions down to 10 fg/μL, corresponding approximately to two genome-equivalent copies of *Salmonella* spp. Each dilution was tested in triplicate. qPCR efficiency (E) was calculated from the standard curve using the equation: E = 10^(−1/slope) − 1.

### Fitness for use of the developed method

The use of real samples allowed verification of the method’s suitability for its intended purpose. To evaluate the test’s ability to operate under different production environments, real samples were collected from 13 broiler chickens’ farms and analyzed. In more details, only paddocks guesting young animals (from 0 to 57 days of age) were sampled. Sampling was carried out using fecal collection boxes: five boxes/flock positioned at the four corners and in the center of each house. The boxes used consisted of a plastic tray covered by a metal mesh. The farms were in the province of Forlì, in the Emilia-Romagna region, Italy, an area with a strong poultry farming tradition and a high density of poultry farms.

These samples are likely to contain highly variable bacterial loads and diverse microflora compositions, reflecting the specific management practices and environmental conditions of each farm. A total of 96 environmental samples were collected from the poultry farms included in the study. On average, five samples were obtained per farm. For farms A and B, two areas containing five specimens each were sampled. One farm (farm A) was sampled at two distinct time points (26 and 40 days post-hatching).

**Figure 1:**
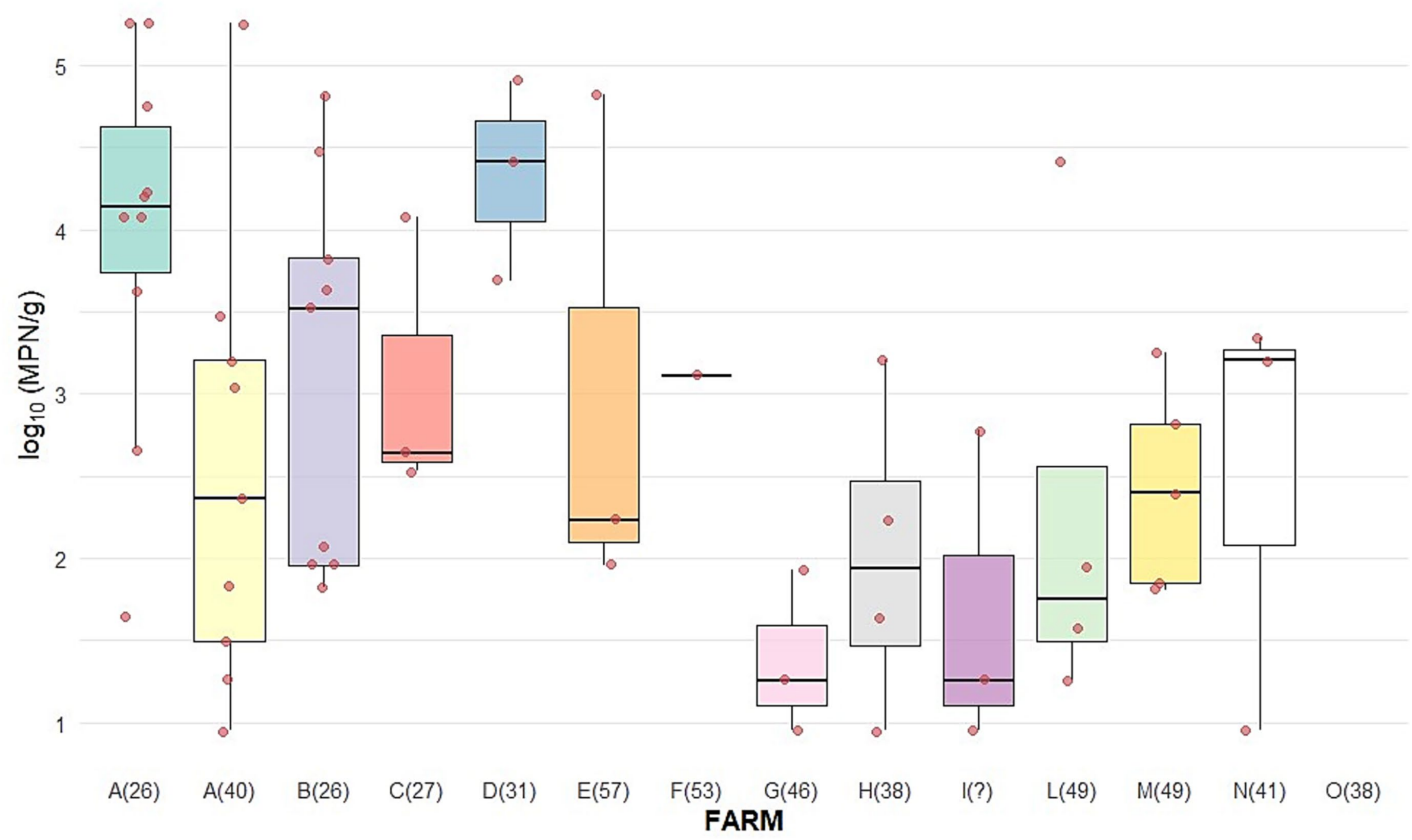
Distribution of values across farms. Boxplots show the median, interquartile range (IQR), and range of values for each farm. The spread of values within each boxplot indicates within-farm variability, while differences in both central tendency and dispersion among farms highlight inter-farm variability. Red dots indicate the actual values of every single measure.

Statistical analysis was performed using SPSS v27 (IBM). Differences in values among farms were assessed using a one-way analysis of variance (ANOVA), with farm as a fixed factor. Homogeneity of variances was evaluated using Levene’s test. Given the relatively balanced sample sizes and the robustness of ANOVA to moderate departures from homoscedasticity, the analysis was considered appropriate. However, due to heterogeneity of variances and the presence of a group with zero variance, the results were further confirmed using the non-parametric Kruskal–Wallis test. Data distribution within and between farms was visually assessed using boxplots (Figure 1).

## Results

### Limits of detection and quantification

According to ISO/TS 6579–2:2012, the limit of detection (LOD) was fixed at one CFU/g of feces. However, considering the number of dilutions and replicates, as well as the analysed volumes in the method described here, the theoretical LOD for this method was estimated at 8.9 CFU/g (0.95 log_10_ CFU/g) corresponding to one positive well at the undiluted level. Moreover, when the Excel sheet provides a numerical value, the method demonstrates not only the detection of the pathogen but also its ability to generate a probabilistic estimate of its concentration. Consequently, negative results cannot be expressed as 0 CFU/g but, according to the concept described above, should be reported as < 0.95 log_10_ CFU/g, indicating that the pathogen, if present, is at a concentration below the detection capability of the method.

In order to define the limit of quantification (LOQ), a series of comparative experiments was performed by testing in parallel two different experimental conditions previously described in the Materials and Methods.

**Figure 2:**
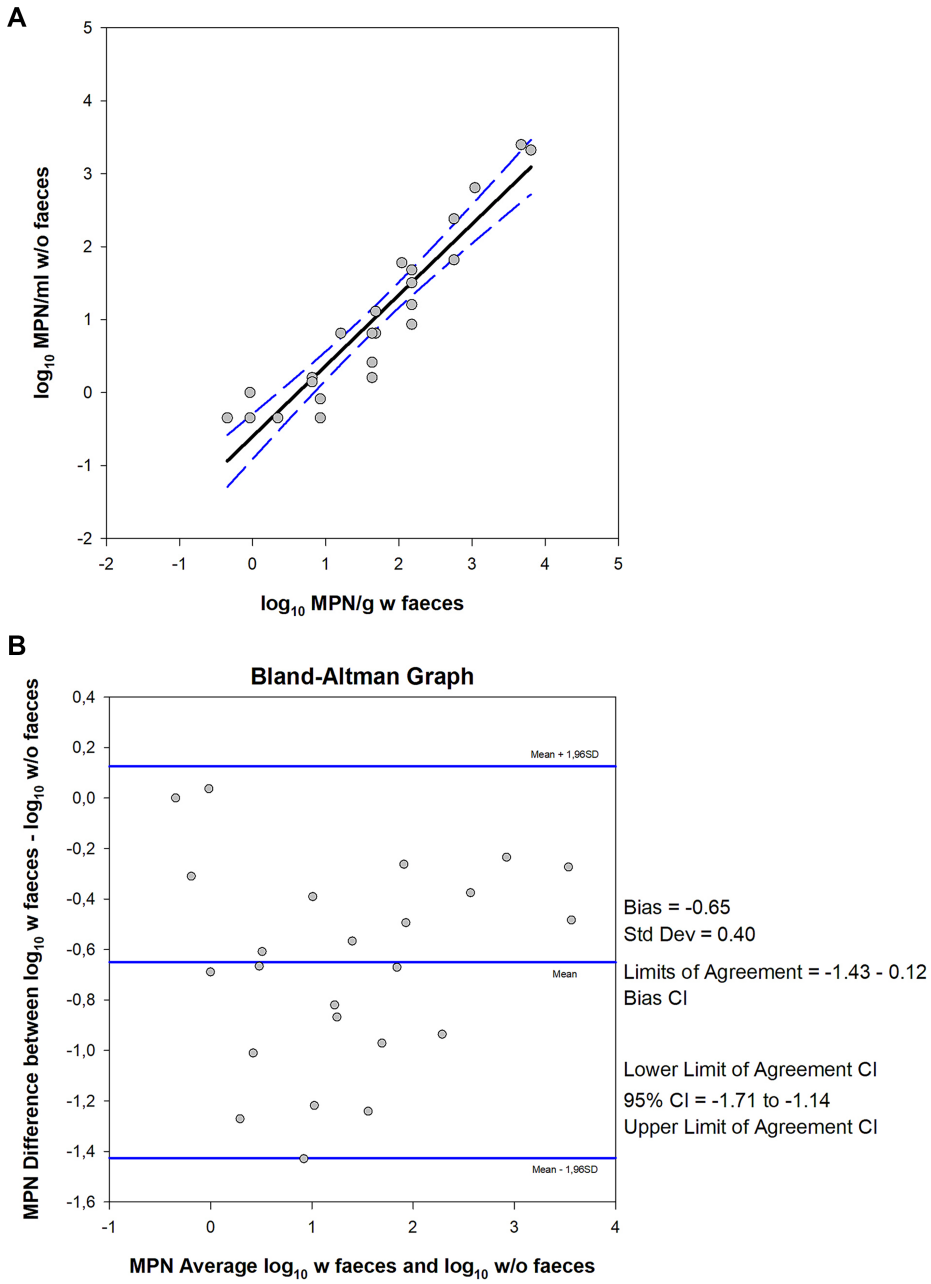
Application of the Bland–Altman method to assess the effect of the sample matrix on the limit of quantification. **(A)** Correlation between the quantification of *Salmonella enterica* subsp. *enterica* serovar Infantis (*S. infantis*) in the presence and absence of fecal material. **(B)** Bland–Altman plot illustrating the agreement and distribution of *S. infantis* loads across the different sample preparations (*n* = 25).

Feces represent an extremely complex matrix, rich in substances such as polysaccharides, bile salts, and particulate debris, which may interfere both with bacterial viability during the early enrichment phase and with the efficiency of qPCR. The results showed a loss of sensitivity of approximately one order of magnitude (one log₁₀): under the same initial contamination level, when the system in BPW detected a bacterial load of three log_10_ (10^3^ CFU/g), the same strain in the fecal matrix was quantified at values close to two log_10_ CFU/g (10^2^ CFU/g, see Figures 2A,B).

This inhibition demonstrates how the fecal matrix can mask the presence of the pathogen, effectively increasing the detection threshold compared with a culture-based sample without matrix interference. In order to visualize this discrepancy and assess the robustness of the method despite the inhibitory effect, the Bland–Altman method was applied.

The resulting Bland–Altman plot allowed the quantification of this bias (systematic deviation), with the points representing matrix replicates were consistently positioned below the zero-line relative to the expected values obtained in BPW (see Figure 2). In more details, this matrix introduced a bias of 0.64 log₁₀ units (standard deviation: 0.40 log₁₀). However, since this deviation remained constant and predictable within the confidence limits, the method was considered valid for diagnostic use.

Despite the approximately one log₁₀ loss in sensitivity, the final limit of quantification (LOQ) remained consistent with the 8.9 CFU/g threshold required by ISO/TS 6579–2:2012, ensuring that the method is able to quantify the bacterium even when the matrix exerts its maximum inhibitory effect.

Notably, the robustness of this result derived also by the use of one reference strain and two field isolates.

### Analytical specificity of the assay

All *Salmonella* Infantis field isolates yielded positive by qPCR, whereas no amplification was detected for isolates belonging to other serovars or to other bacterial species.

### Repeatability and reproducibility

Repeatability results are reported in Table 1 for both spiked and naïve samples and are expressed as log₁₀ values. The coefficient of variation (CV) was below 10% across all conditions, suggesting a good repeatability of the method.

Reproducibility results are reported in Table 2 for both spiked and naïve samples, with CV values consistently remaining below 10%, with the only exception of 2 log_10_ for spiked samples where the CV resulted around 20%.

**Table 2:** Reproducibility of spiked **(A)** and naturally contaminated samples **(B)**.

(A) Spiked samples					
Concentration (MPN/g feces)	Rep 1 (log_10_ MPN/g)	Rep 2 (log_10_ MPN/g)	Mean	SD	CV (%)
3 log_10_	3.23	3.20	3.22	0.02	0.6
2 log_10_	1.85	2.51	2.18	0.47	21.5
1 log_10_	1.72	1.96	1.84	0.17	9.4

(B) Real samples					
Samples	Rep 1 (log_10_ MPN/g)	Rep 2 (log_10_ MPN/g)	Mean	SD	CV (%)
1	4.54	4.23	4.39	0.22	5.1
2	2.77	2.77	2.77	0.00	0.0

### Fitness for use of the developed method

To validate the method under conditions representative of real epidemiological scenarios, 96 environmental samples were analyzed. Quantification results exhibited considerable variability, ranging from below the limit of detection (< 0.95 log₁₀ MPN/g of feces) to 5.25 log₁₀ MPN/g of environmental feces (Figure 1). One farm (farm O) yielded no positive results. The single farm sampled at two time points showed a decrease in median concentration from 4.14 log₁₀ MPN/g at 26 days post-hatching to 2.70 log₁₀ MPN/g at 40 days post-hatching.

Figure 1 illustrates the distribution of values within and between farms. Within-farm variability was evident from the spread of values within individual farms, as shown by the interquartile ranges and overall dispersion of the data. Levene’s test indicated significant heterogeneity of variances across farms (*p* < 0.05), suggesting that within-farm variability was not homogeneous. Nevertheless, a one-way ANOVA revealed a significant effect of farm on the measured values (*F* = 3.26, *p* < 0.001), indicating that variability between farms exceeded that observed within farms. These findings were supported by the visual differences among farms in Figure 1, where both the central tendency and dispersion varied across groups. Consistent results were obtained using the Kruskal–Wallis test (*p* = 0.002), confirming the robustness of the observed differences among farms.

Collectively, these findings indicate that the method is fit for purpose for quantifying the load of *S. infantis* in environmental fecal specimens.

## Discussion

The first detection of a *Salmonella* Infantis multidrug-resistant ESBL-producing clone in Italian broiler chickens occurred in 2011, which subsequently led to human infections in 2013–2014 (Franco et al., 2015), clearly illustrates the direct farm-to-fork transmission pathway in the Italian context. Furthermore, a study conducted in Central Italy confirmed that multidrug-resistant (MDR) *S. infantis* harboring pESI-like plasmids carrying blaCTX-M-1 genes was detected at multiple sampling points along the food chain, with a prevalence of 97% among the samples investigated (Russo et al., 2024). Finally, surveillance data from 2016 to 2019 documented high rates of resistance to nalidixic acid, tetracycline, and sulphamethoxazole/trimethoprim among Italian *S. infantis* field isolates (Proietti et al., 2020), confirming the persistence and dissemination of MDR *S. infantis* throughout the broiler meat production chain in Italy.

From an industry perspective, *S. infantis* poses significant economic challenges, including increased monitoring costs, potential trade restrictions, flock depopulation requirements, and reduced consumer confidence in poultry products. The need for enhanced biosecurity measures, specialized cleaning and disinfection protocols, and prudent antimicrobial stewardship further increases operational complexity and costs in production systems that already operate under tight margins. In Italy, where poultry production represents a substantial component of the agricultural economy, the high prevalence and antimicrobial resistance profile of *S. infantis* present additional challenges for maintaining both domestic and international market competitiveness while ensuring food safety.

Controlling *S. infantis* at farm level is challenging and includes many different approaches. Such as vaccination, proper litter cleaning and disinfection, use of feeds supplemented with pre/pro/post/syn biotics, use of antibiotics or bacteriophages and water sanitation, although water contamination by *Salmonella* spp. is typically minimal (Obe et al., 2023; Betiku et al., 2025). Moreover, no single measure can fully eliminate the risk of *Salmonella* spp. on poultry farms, and instead multi-hurdle, multi-level strategies are better suited to reducing Salmonella loads in these settings (Kimminau et al., 2021; Betiku et al., 2025). In this contest, vaccination is a safe and effective way of protecting animals against diseases and different research groups are currently developing and evaluating an effective vaccine against *S. infantis* in poultry production (Tolooe et al., 2025). Currently in Italy, there is one authorized vaccine against *S. infantis*, which is an inactivated trivalent bacterin, targeting *S. enteritidis*, *S. typhimurium* and *S. infantis* (Nobilis® Salenvac ETC produced by MSD Animal Health). This vaccine is administered by injection during the rearing phase of broiler breeders’ production (Crouch et al., 2020).

Irrespective of the approach employed, it’s clear that a reduction in the environmental loads of *Salmonella* spp., and vice versa, is pivotal in reducing the probability of recovering the pathogen (Gole et al., 2014). Based on this concept, the ability of diagnostic tools able not only of detecting but also on quantifying the load of *S. infantis* can be pivotal for measuring if these strategies are working.

The MPN approach, combined with molecular confirmatory methods, has proven effective for quantifying pathogens across different matrices. It has been widely applied in water microbiology, including the detection of *Salmonella* spp. (Jenkins et al., 2008), *Escherichia coli*, and Gram-positive *Enterococcus faecalis* (Ahmad et al., 2017). This same combined methodology has also been extended beyond water analysis. For example, it has been successfully used to enumerate *Salmonella* spp. in food samples (Shashidhar et al., 2011) and *Listeria monocytogenes* (De Martinis et al., 2007). Additionally, in environmental swab sampling, the MPN approach coupled with molecular confirmation has enabled effective enumeration of *Bacillus anthracis* (Létant et al., 2010).

Overall, these studies highlight the versatility of the MPN–molecular detection strategy for pathogen quantification in diverse sample types, including water, food, and environmental surfaces.

The environmental concentration of *Salmonella* spp. in poultry litter can vary considerably. A recent study of 13 farms in the USA found concentrations ranging from <0.45 to >280,000 MPN/g (Dunn et al., 2022), while another study analyzed litter samples from 18 broiler farms across three seasons (spring, summer and winter), reporting a geometric mean of 0.21 ± 20.7 MPN/g (Gutierrez et al., 2020), essentially confirming the high variability of environmental samplings. Our preliminary observations, focusing exclusively on *S. infantis*, indicate a high degree of variability in its environmental load, both within and between farms.

The method developed in this study can detect environmental *S. infantis* loads ranging from less than 10 MPN/g of feces to over infinity in a robust manner. Moreover, according to our data, it is well suited to this purpose (see Figure 1) and could be represent a tool to evaluate the dynamics of the infection during the production cycle, to understand the causes of the introduction and persistence of *S. infantis* in meat-poultry production and, finally, to evaluate (in combination with feedback data from the slaughterhouse) the effectiveness of the prevention measures adopted to control the infection.

At the same time, we recognize that, to effectively combat *Salmonella* Infantis. in the poultry industry, greater emphasis should be placed on a multi-intervention strategy that reduces the bacterial load in birds and ultimately prevents carcass contamination at processing plants, especially in the current era of multidrug-resistant Salmonella (Shaji et al., 2023).

## Data Availability

The raw data supporting the conclusions of this article will be made available by the authors, without undue reservation.

## Supplementary material

SUPPLEMENTARY FIGURE 1 Efficiency of the qPCR assay used in this study. The upper graph was generated by analyzing, in triplicate, pure DNA from *S. infantis* NCTC 6703/IZSLER 2150 at each concentration point. The lower graph was generated by analyzing, in triplicate, pure DNA from a *S. infantis* field isolate at each concentration point.
