# Supplementary figures and images for: Development and validation of a quantitative method for the enumeration of Salmonella enterica serovar Infantis from environmental poultry feces based on most probable number approach followed by confirmatory qPCR
Source: Front Microbiol. 2026 Jun 18;17:1861550. doi: 10.3389/fmicb.2026.1861550 (PMC13323025; doi:10.3389/fmicb.2026.1861550)

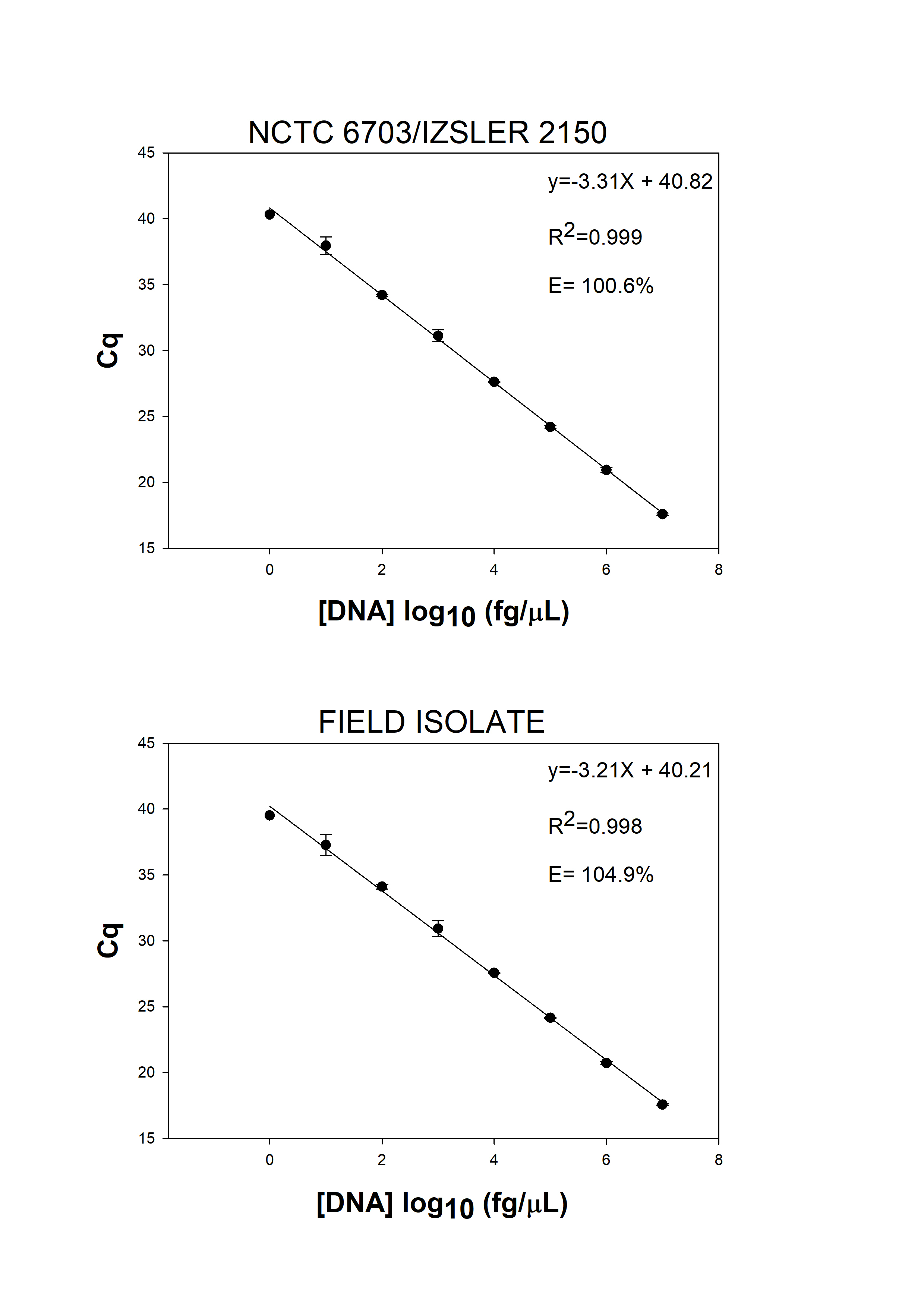

Supplement: SUPPLEMENTARY FIGURE 1 — Efficiency of the qPCR assay used in this study. The upper graph was generated by analyzing, in triplicate, pure DNA from S. infantis NCTC 6703/IZSLER 2150 at each concentration point. The lower graph was generated by analyzing, in triplicate, pure DNA from a S. infantis field isolate at each concentration point. [file Image_1.jpeg]
