# Supplementary material for: Development and validation of a quantitative method for the enumeration of Salmonella enterica serovar Infantis from environmental poultry feces based on most probable number approach followed by confirmatory qPCR
Source: Front Microbiol. 2026 Jun 18;17:1861550. doi: 10.3389/fmicb.2026.1861550 (PMC13323025; doi:10.3389/fmicb.2026.1861550)
Supplement: Supplementary file 2 [file Data_Sheet_1.pdf]

Table 1 Supplemental material

List of *Salmonella enterica* subsp. *enterica* belonging to different serovars than *S*. Infantis and other bacteria used for testing the exclusivity

Serovar	Number of field isolates tested	ID (year 2022-2023)
*Salmonella S*oftener	2	14251/1, 399558/1
Salmonella Veneziana	2	255326/1, 137373
Salmonella Pullorum	1	296267
Salmonella Coeln	1	286488
Salmonella Takoradi	1	52286
Salmonella Hessarek	1	110587
Salmonella Newport	1	320146
Salmonella Kentucky	3	14103, 25009, 27767
Salmonella Zaiman	1	256011
Salmonella Farmingdale	1	272395
Salmonella Blockley	1	328050
Salmonella Agama	2	52386, 218891
Salmonella Enteritidis	2	281798, 24424
Salmonella Typhimurium	7	248044, 13829/1, 13829/5, 18080, 21348,21361, 24544
Salmonella Mikawasima	1	255121
Salmonella Rissen	1	296267
Salmonella Litchfield	1	253876/3
Salmonella Tennessee	1	379504
Salmonella Bredeney	2	175068/5, 122921
Salmonella Anatum	3	159075, 182286/5, 268195/1
Monophasic Salmonella Typhimurium *(1,4,[5],12:i:-)*	9	59866, 20092, 22276, 30515, 30955, 34829, 35053/1, 35053/2, 35965
Salmonella Carno	1	300582/5
Salmonella Agona	3	217139/1, 17027, 35053
Salmonella Derby	2	331475/5, 30518
Salmonella Dublin	1	15327, 25647/2
Salmonella Goldcoast	1	28818
Salmonella Cholerasuis	5	18994, 22299, 24684, 30288, 32282/2
Salmonella Mishmarhaemek	1	27698
Salmonella Napoli	1	34838
Salmonella Senftenberg	1	27764
Escherichia. coli	2	ATCC 12923, 25922
Citrobacter freundii	1	ATCC 43864
Proteus mirabilis	1	29906
Listeria monoctyogenes	26	9915/1, 9915/2, 17543/1, 17543/2, 17543/3, 17543/4, 175432/5, 24239/1, 24239/2, 24239/3, 24239/4, 24239/5, 431735/3, 431735/5,13646/1,13646/2, 13646/3, 13646/4, 13646/5, 13693, 14001/1, 14001/2, 14001/4, 14001/6, 14001/7
