# Supplementary material for: Development and validation of a quantitative method for the enumeration of Salmonella enterica serovar Infantis from environmental poultry feces based on most probable number approach followed by confirmatory qPCR
Source: Front Microbiol. 2026 Jun 18;17:1861550. doi: 10.3389/fmicb.2026.1861550 (PMC13323025; doi:10.3389/fmicb.2026.1861550)
Supplement: Supplementary file 3 [file Table_2.docx]

# Table 2 (Supplementary Material). Median, interquartile range, minimum, maximum, and positive ratio of environmental samples analyzed for the presence of *S*. Infantis.

|  | **FARM ID (Log_10_ MPN/g f** **environmental faeces)** | | | | | | | | | | | | | |
| --- | --- | --- | --- | --- | --- | --- | --- | --- | --- | --- | --- | --- | --- | --- |
|  | **A(26)** | **A(40)** | **B(26)** | **C(27)** | **D(31)** | **E(57)** | **F(53)** | **G (46)** | **H (38)** | **I (?)** | **L (49)** | **M (49)** | **N (41)** | **O (38)** |
| **Median** | 4.14 | 2.36 | 3.52 | 2.64 | 4.41 | 2.23 | 3.11 | 1.26 | 1.94 | 1.26 | 1.75 | 2.40 | 3.20 | 0 |
| **25^th^ inter.** | 3.74 | 1.49 | 1.96 | 2.59 | 4.05 | 2.09 | 3.11 | 1.10 | 1.47 | 1.10 | 1.49 | 1.85 | 2.08 | 0 |
| **75^th^ inter.** | 4.62 | 3.20 | 3.83 | 3.36 | 4.66 | 3.52 | 3.11 | 1.59 | 2.47 | 2.02 | 2.56 | 2.81 | 3.27 | 0 |
| **max** | 5.26 | 5.26 | 4.82 | 4.08 | 4.91 | 4.82 | 3.11 | 1.92 | 3.20 | 2.78 | 4.41 | 3.26 | 3.34 | 0 |
| **min** | 1.64 | <0.95 | 1.83 | 2.53 | 3.69 | 1.96 | <0.95 | <0.95 | <0.95 | <0.95 | <0.95 | 1.81 | <0.95 | 0 |
| **positive ratio*** | 10/10 | 9/10 | 9/10 | 3/5 | 3/5 | 3/5 | 1/5 | 3/5 | 4/5 | 3/5 | 4/5 | 5/5 | 3/5 | 0/5 |

* Samples successfully quantified vs. total samples analyzed
